# Multimodal Flotation Sensing: A Systematic Review of State Identification and Sensor Readiness

**DOI:** 10.3390/s26175560

**Published:** 2026-09-01

**Authors:** Karshyga Akishev, Alexandr Podvalov, Abdikarim Zeinullin, Yelaman Aibuldinov, Arman Nurmaganbetov, Nursultan Toktar, Sabina Khussainova

**Affiliations:** 1 Department of Computer Engineering and Automation, K. Kulazhanov Kazakh University of Technology and Business, Astana 010000, Kazakhstan; akmail04cx@mail.ru; 2 AG TECH LLP, Astana 010000, Kazakhstan; a.podvalov@ag-tech.kz (A.P.); a.nurmaganbetov@ag-tech.kz (A.N.); sabina.maratovna@gmail.com (S.K.); 3 Department of Chemistry and Chemical Technologies, K. Kulazhanov Kazakh University of Technology and Business, Astana 010000, Kazakhstan; karim_57@mail.ru; 4 Office of the Vice-Rector for Science and Innovation, K. Kulazhanov Kazakh University of Technology and Business, Astana 010000, Kazakhstan; elaman_@mail.ru; 5 EL Invest Research and Production Company LLP, Astana 010000, Kazakhstan; 6 Faculty of Energy, Toraighyrov University, Pavlodar 140008, Kazakhstan

**Keywords:** froth flotation, intelligent sensing, machine vision, froth image analysis, multimodal data fusion, soft sensor, process state identification, industrial automation, sensor readiness

## Abstract

Reliable state identification is essential for intelligent flotation control because recovery, concentrate grade, entrainment, and mineral losses are only partially observable online. This systematic review examines field instrumentation, online analyzers, froth imaging, temporal synchronization, machine-vision methods, multimodal soft sensing, and the engineering requirements that determine whether a predictive model can operate as an industrial sensor. Scopus and Web of Science publications from 2021 to June 2026 were screened using a PRISMA-based protocol. The systematic evidence base includes 98 peer-reviewed technical studies published between 2021 and June 2026, and two PRISMA methodological publications are used to ensure the methodology for presenting the review. Additional methodological and contextual sources cited outside the systematic body of evidence are not included in the number of studies reflected in PRISMA. The evidence shows that machine vision is the most mature non-contact sensing approach, supporting bubble-size measurement, froth-velocity estimation, operating-state recognition, grade prediction, and visual monitoring. Current research is shifting from handcrafted descriptors toward convolutional, transformer, self-supervised, graph-based, temporal, and multimodal models. However, predictive accuracy alone does not demonstrate industrial readiness when camera geometry, illumination, contamination, delay compensation, temporal leakage, domain shift, uncertainty, inference latency, and SCADA/PLC integration are not evaluated. A five-dimensional Sensor Readiness Index is proposed to assess metrological validity, temporal integrity, validation rigor, operational robustness, and automation integration. The review defines the principal requirements for reliable industrial deployment of flotation sensing systems.

## 1. Introduction

This systematic review was designed and reported in accordance with the PRISMA 2020 statement and its explanation and elaboration document [1,2]. The scope is limited to measurement, sensing, state identification, and the supporting information architecture of flotation systems. Optimization and control are considered only where they impose explicit requirements on sensor outputs, such as latency, uncertainty, validity flags, and integration with supervisory automation.

Froth flotation is a nonlinear, multivariable separation process in which recovery, concentrate grade, mass pull, and tailings losses are jointly influenced by feed mineralogy, particle-size distribution, liberation, pulp chemistry, gas dispersion, reagent dosage, froth depth, equipment condition, and the interaction between pulp and froth phases. Recent reviews have summarized control-oriented flotation models, froth-image analysis, machine-learning applications, deep-learning pipelines, multidimensional visual features, and the organizational barriers that constrain data-driven deployment [3,4,5,6,7,8,9]. These publications demonstrate strong growth of artificial intelligence in mineral processing, but they also reveal a persistent division between three research traditions: process modelling, computer vision, and industrial automation. The first tradition emphasizes dynamic equations and control performance; the second emphasizes image metrics and predictive accuracy; the third emphasizes sensors, data infrastructure, maintainability, and operator interaction.

The measurement problem is more fundamental than the selection of a learning algorithm. Direct online measurement of the principal metallurgical outputs remains difficult. Laboratory assays are accurate but delayed; online X-ray fluorescence and spectroscopic analyzers require sampling and calibration infrastructure; field instruments primarily describe hydrodynamic and chemical conditions; and cameras provide high-frequency indirect observations of the froth surface. Recent work on digital laboratories, physicochemical prediction, soft sensing, data-quality control, explainable modelling, and public image datasets confirms that the final estimate depends on the quality and temporal coherence of the entire observation chain rather than on a single sensor or model [10,11,12,13,14,15]. A froth image is therefore not automatically a measurement. It becomes a measurement only when the imaging geometry, illumination, calibration or verification procedure, timestamp, quality state, output definition, and uncertainty are specified.

Machine vision is currently the most developed non-contact sensing direction in flotation. Between 2021 and 2026, studies progressed from multivariate image descriptors and conventional classification toward semantic representation learning, attention-based monitoring, temporal grade estimation, ash-content prediction, transformer models, instance and semantic segmentation, velocity estimation, multimodal hypergraphs, self-supervised representation learning, ensemble sensing, and industrial performance assessment [16,17,18,19,20,21,22,23,24,25,26,27,28,29,30,31,32,33,34,35,36,37,38,39,40,41,42,43,44,45,46,47,48,49]. The diversity of these tasks has created a new methodological problem: results expressed as accuracy, $R^{2}$, RMSE, intersection-over-union, or mean velocity error are often compared without considering whether the camera was stable, whether frames from the same video interval were separated across training and test sets, whether the prediction was validated on a different campaign, and whether the system produced a quality-qualified signal within the permissible control cycle.

Soft sensors and multimodal models extend the observation system beyond images. Recent studies estimate concentrate grade, tailings grade, silica impurity, sulfur removal, recovery, ash content, and other delayed variables from combinations of process measurements, froth images, Raman spectra, temporal models, physics-informed structures, transfer learning, and cross-feature transformers [50,51,52,53,54,55,56,57,58,59,60,61,62,63,64,65,66,67,68,69,70]. These studies confirm the value of combining process and visual data, but they also show that the target variable is commonly defined by delayed or composite laboratory samples. As a result, label alignment and uncertainty are not secondary preprocessing details; they determine what physical state the model is actually learning.

The industrial sensing layer must also coexist with dynamic models, digital twins, control loops, actuator limits, and supervisory decision logic. Recent work addresses intelligent monitoring and optimization, digital-twin dosing, adaptive surrogate models, physics-informed forecasting, predictive control, level-control implementation, Bayesian tuning, multiobjective optimization, reagent design, and physics-informed grade prediction [71,72,73,74,75,76,77,78,79,80,81,82,83,84]. These studies are not treated here as a separate control review. Instead, they are used to derive requirements for the sensor layer: an estimate must be available at the right time, be valid within a stated operating domain, include uncertainty or a confidence indicator, and remain traceable from acquisition through data processing to the supervisory system.

The rapid development of computer vision and time-series learning provides useful methodological tools, including vision transformers, shifted-window architectures, self-supervised learning, masked pretraining, promptable segmentation, modern convolutional networks, video attention, vision-language pretraining, uncertainty quantification, anomaly detection, domain generalization, transformer-based time-series representation, long-horizon forecasting, and probabilistic imputation [85,86,87,88,89,90,91,92,93,94,95,96,97,98,99,100]. Their transfer to flotation is promising but cannot be assessed solely by architecture novelty. The physical measurement chain and industrial validation strategy remain decisive.

The present review treats field instruments, cameras, online analyzers, laboratory measurements, equipment feedback, and data-driven estimates as components of a unified multimodal observation system. The objective of this systematic review is to establish an integrated methodological framework for flotation state identification and to determine the conditions under which data-driven models can be regarded as reliable, automation-ready industrial sensors. The review classifies measurement channels according to the measurand, temporal behavior, sources of uncertainty, and their role in state identification; critically compares recent machine-vision and multimodal sensing methods; distinguishes algorithmic performance from industrial sensor readiness; and formulates a reporting, validation, and deployment framework for reproducible flotation sensing. The review is independent and self-contained.

The scientific novelty of this systematic review lies in the integrated evaluation of flotation sensing as a complete measurement-to-automation chain rather than as a collection of isolated instruments or predictive algorithms. Unlike previous reviews focused primarily on froth-image features, model architectures, or predictive accuracy, the present study jointly examines physical, chemical, visual, spectroscopic, and analytical measurement channels together with their temporal alignment, validation strategy, uncertainty, operational robustness, and industrial interfaces. A five-dimensional Sensor Readiness Index is proposed to distinguish metrological validity, temporal integrity, validation rigor, operational robustness, and automation integration. The proposed SRI is not intended to replace general technology maturity models, such as the Technology Readiness Level (TRL). TRL characterizes the degree of technology maturity and the level of its demonstration in progressively more representative operating conditions, whereas SRI addresses a narrower task: it determines whether there is a sufficient set of metrological, temporal, validation, operational, and integration evidence to allow a data-based model to be considered a reliable industrial sensor. Therefore, TRL and SRI are complementary, not interchangeable, assessment tools [101]. In addition, a four-level evidence framework and a staged industrial validation sequence are introduced to differentiate laboratory feasibility, campaign-level validation, external transferability, and maintained online sensing. This synthesis establishes explicit criteria for determining when a data-driven flotation model can be regarded as an industrial sensor rather than only an image-analysis or prediction algorithm.

The following research questions guide the synthesis:

**RQ1.** Which physical, chemical, visual, spectroscopic, and analytical measurement channels are used for flotation state identification, and what temporal limitations characterize them?

**RQ2.** Which machine-vision and learning methods are used to measure bubble structure, froth motion, texture, operating state, and metallurgical performance?

**RQ3.** How are heterogeneous data streams, transport delays, laboratory labels, and temporally correlated video observations synchronized and validated?

**RQ4.** Which criteria distinguish a laboratory image-analysis model from a robust industrial visual sensor?

**RQ5.** Which research gaps currently prevent continuous, transferable, uncertainty-aware, and automation-ready sensing in full-scale flotation plants?

## 2. Flotation as a Multimodal Sensing Problem

### 2.1. Observable and Latent Process States

**Figure 1:**
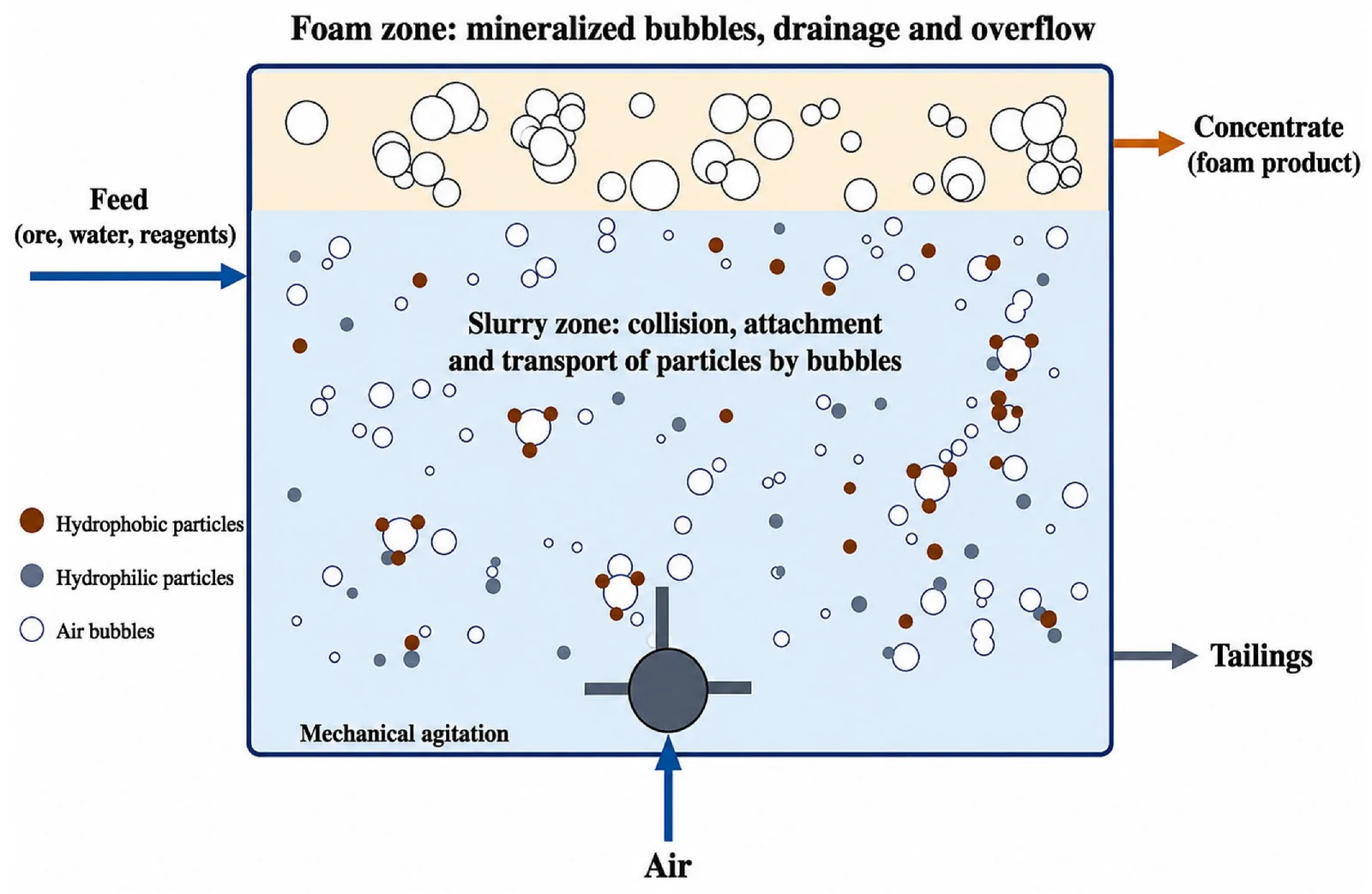
Schematic representation of the froth flotation process.

Figure 1 illustrates the physical structure of a flotation cell and identifies the pulp and froth regions from which process and visual measurements are obtained. Feed properties act mainly as disturbances, whereas airflow, pulp level, reagent dosage, and feed rate influence the operating regime. Concentrate and tailings assays describe metallurgical performance, but bubble mineralization, attachment probability, gas holdup, drainage rate, mineral loading, and effective froth stability remain latent.

A unified observation vector can be represented as (1):

(1) $$z_{k}={\left[s_{k}^{T},\;\phi {\left(I_{k}\right)}^{T},\;a_{k}^{T},\;q_{k}^{T}\right]}^{T},$$

where $s_{k}$ is the vector of process measurements available at discrete time k, $I_{k}$ is the acquired froth image or video frame, ϕ(⋅) is the visual-feature operator, $a_{k}$ contains online or laboratory analytical results, and $q_{k}$ contains data-quality, equipment-state, and channel-health indicators. The inclusion of $q_{k}$ is essential: a stale analyzer value, a camera with a contaminated window, and a valid measurement should not enter the model as equivalent numerical inputs.

The observation vector does not represent a single physical instant merely because all elements have the same database timestamp. Each channel has its own acquisition, transport, filtering, analysis, and reporting delay. The same is true for labels: a concentrate assay may represent a composite sample collected over several minutes, while a froth image represents a narrow exposure interval. A sensor model that ignores this distinction may learn correlations between non-equivalent process states.

Table 1 summarizes the principal measurement channels, their characteristic delays and risks, and their roles in flotation state identification.

### 2.2. Temporal Alignment and Label Definition

Multirate data are a defining feature of industrial flotation. A camera may produce 10–30 frames per second, a PLC may archive selected tags each second, an online analyzer may produce one result every several minutes, and a laboratory assay may represent a composite sample collected over a longer interval. Directly joining these records by nearest timestamp can create strong but physically false relationships. Transport time from the cell to the sampling point, residence time in launders and pipelines, analyzer cycle duration, and the interval over which a composite sample is accumulated must be considered explicitly.

For channel j, a delay-compensated series can be expressed as (2):

(2) $${\tilde{s}}_{j,k}=s_{j}\left(k-l_{j}\right),\;\;l_{j}=round\;\left(\frac{\tau_{j}}{\Delta t}\right),$$

where $\tau_{j}$ is the effective physical and analytical delay and Δt is the common resampling interval. A fixed delay is only a first approximation. In practice, $\tau_{j}$ may change with flow rate, valve configuration, sampling system status, or operating regime. Consequently, an industrial dataset should preserve the original event time, acquisition time, receipt time, processing time, and the delay model used to construct the training label.

The label should also be defined as an interval rather than an instantaneous point when it originates from composite sampling. Let the assay reported at time $t_{r}$ represent material collected between $t_{s}$ and $t_{e}$. A consistent image or process feature should then be aggregated over an event-time window shifted by the relevant transport delay (3):

(3) $${\bar{z}}_{i}=\frac{1}{N_{i}}\sum_{k\in \Omega_{i}}z_{k},\;\;\Omega_{i}=\left\{k:\;t_{s}-\tau_{tr}\leq t_{k}<t_{e}-\tau_{tr}\right\}.$$

This formulation prevents the common error of assigning one laboratory value to a single arbitrarily selected frame. It also allows robust statistics, such as medians, quantiles, temporal gradients, and variability descriptors, to be computed over the physically relevant interval.

### 2.3. Quality-Aware Data Fusion

Missing values, stale measurements, outliers, and sensor degradation should not be treated as ordinary numerical variation. A quality-aware fusion model receives both the synchronized observations and their quality indicators (4):

(4) $$\left({\hat{x}}_{k},{\hat{y}}_{k+h},\sigma_{k}\right)=F\;\left(Q_{k-L:k}⊙Z_{k-L:k}\right),$$

where $Z_{k-L:k}$ is a temporal window of multimodal observations, $Q_{k-L:k}$ is a quality matrix, ${\hat{x}}_{k}$ is the current state estimate, ${\hat{y}}_{k+h}$ is a predicted output, and $\sigma_{k}$ represents predictive uncertainty. The element-wise operation indicates that unavailable or invalid channels may be masked, down-weighted, or processed through a dedicated missingness mechanism.

Outlier removal based only on statistical distance is particularly risky in flotation. Start-ups, ore transitions, reagent disturbances, temporary froth collapse, sensor cleaning, and operating-mode changes may appear anomalous while containing the most valuable information for robust model development. Data screening should therefore combine statistical methods with operating-mode labels, equipment status, process constraints, and expert review. A suitable record should distinguish at least four classes: physically valid normal operation, physically valid transition, instrumentation fault, and unknown or unverified state.

### 2.4. Industrial Information Architecture

**Figure 2:**
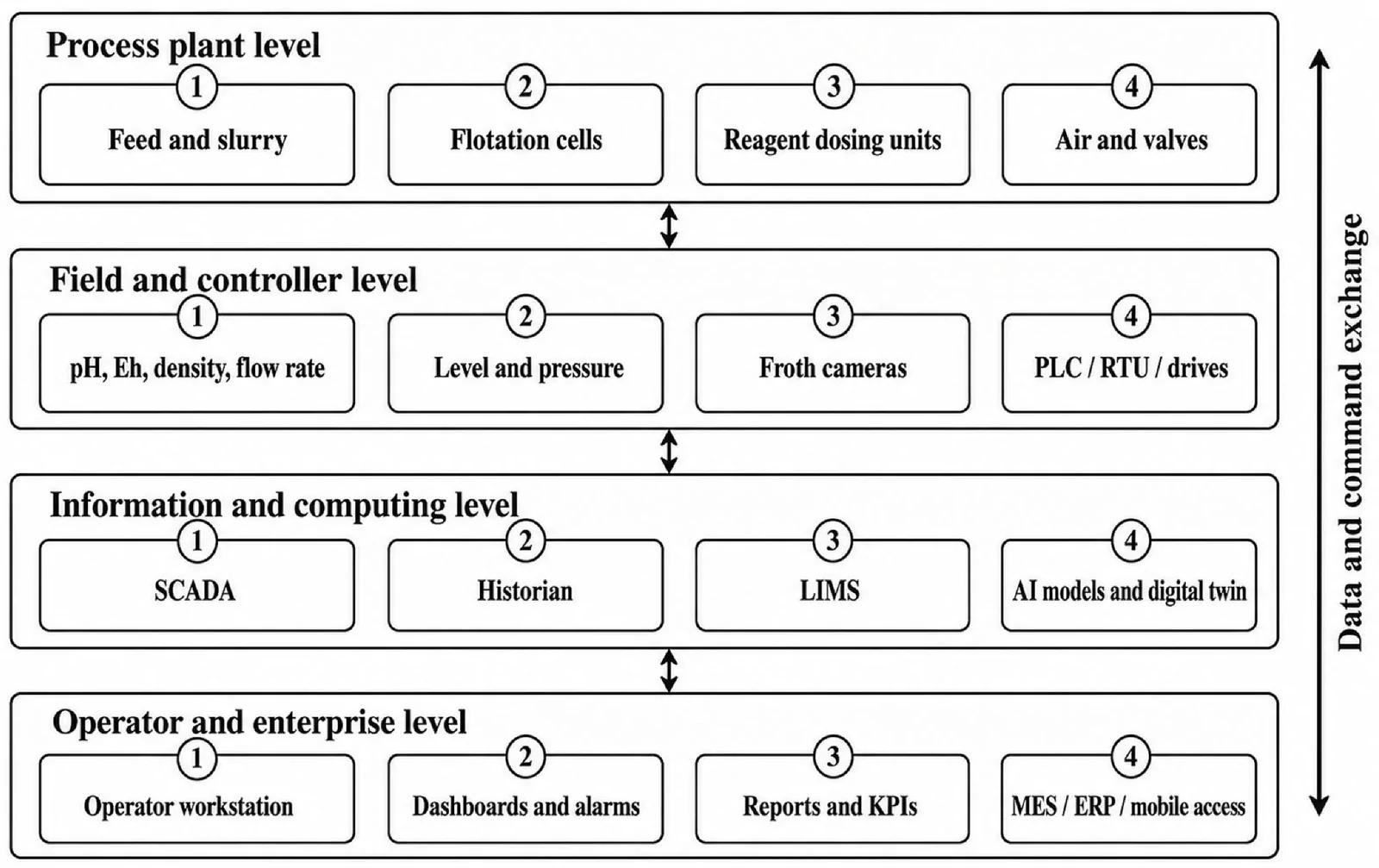
Multi-layer architecture for multimodal flotation sensing and industrial data exchange.

Figure 2 presents the layered automation architecture used to evaluate industrial sensor readiness. It links the flotation equipment and field instrumentation with PLC/SCADA acquisition, historian and LIMS services, AI inference, operator visualization, and enterprise-level data exchange.

The architecture shown in Figure 2 implies five traceability requirements. First, the raw measurement and the processed estimate should retain a common event identifier. Second, the model version, preprocessing configuration, and input quality state should be archived with every prediction. Third, the system should distinguish the AI-generated recommendation, the PLC command, and the actual actuator response. Fourth, an invalid or out-of-domain estimate should be visible to the operator rather than silently replaced by a plausible number. Fifth, time synchronization across camera, PLC, analyzer, historian, and LIMS should be verified rather than assumed.

**Figure 3:**
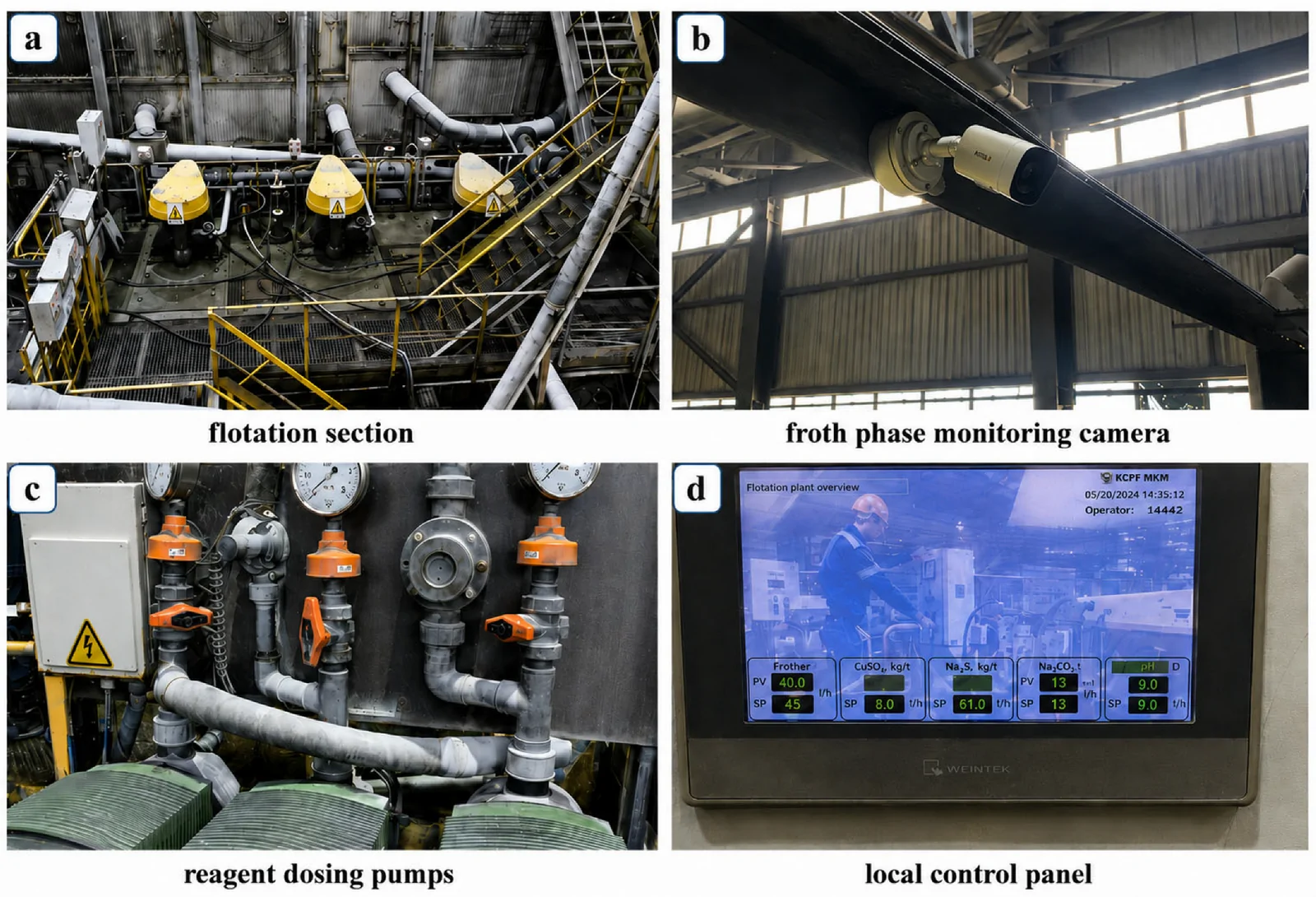
Elements of an industrial flotation installation: (**a**) flotation section; (**b**) froth monitoring camera; (**c**) reagent dosing pumps; and (**d**) local operator control panel (photographs by the authors).

Figure 3 illustrates the industrial conditions that influence sensing reliability, including camera mounting, dust, vibration, ambient-light variation, optical contamination, reagent-dosing dynamics, and local operator interfaces. The photographs provide engineering context and are not treated as an experimental dataset.

## 3. Materials and Methods

### 3.1. Review Design and Search Period

**Figure 4:**
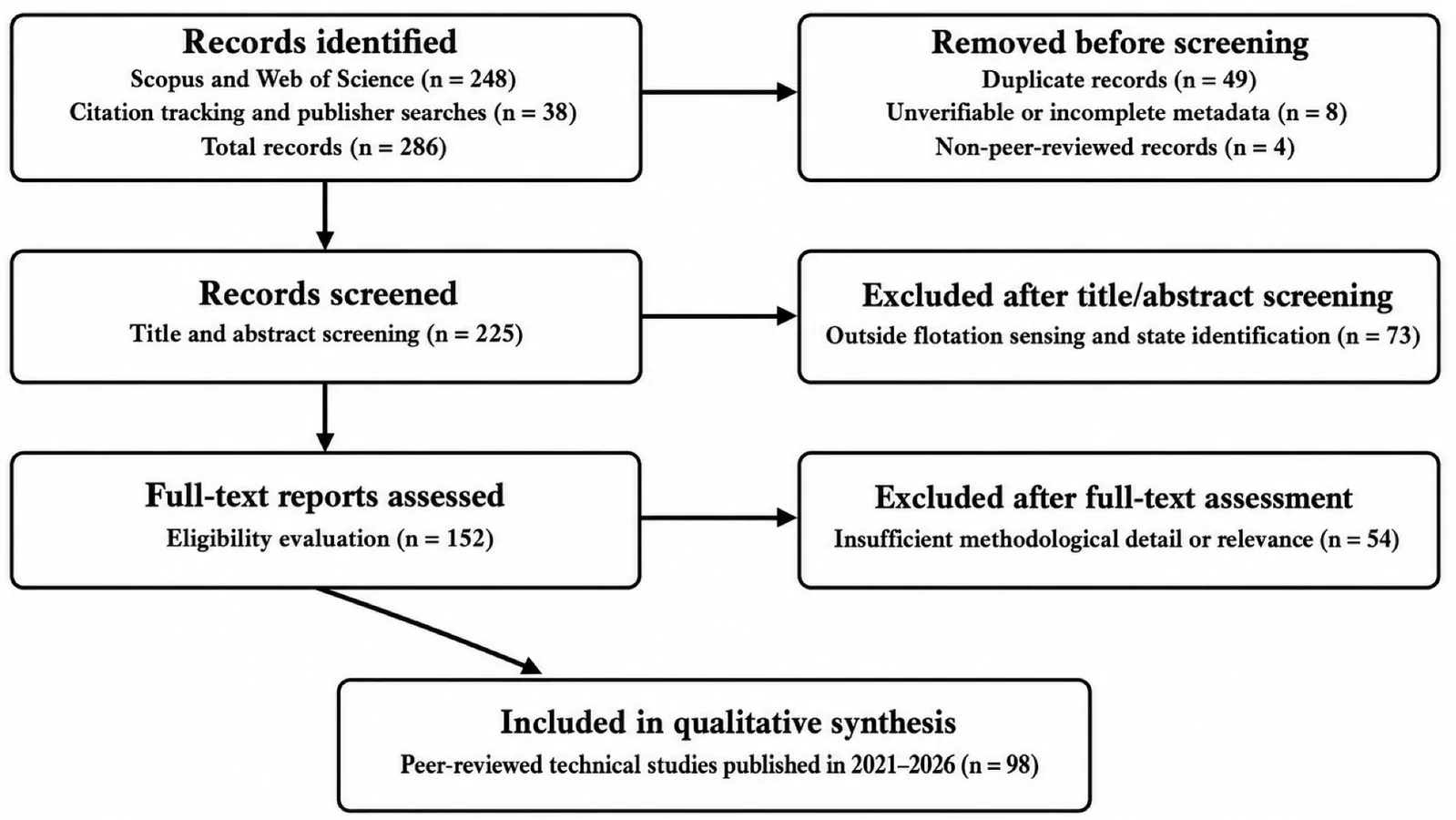
PRISMA-based study-selection workflow for the 2021–2026 evidence base.

This systematic review was designed, conducted, and reported in accordance with the PRISMA 2020 statement and its explanation and elaboration document [1,2]. The study-selection process is summarized in Figure 4; the completed PRISMA 2020 checklist is provided as Supplementary Material. This review was not registered in a public registry (e.g., PROSPERO), and a formal review protocol was not prepared prior to conducting the review.

A systematic critical review with descriptive, comparative, and thematic synthesis was conducted. A quantitative meta-analysis of accuracy, RMSE, $R^{2}$, or segmentation scores was not performed because the reviewed studies differ substantially in ore type, flotation stage, camera configuration, label definition, dataset size, temporal sampling, target variable, and validation strategy. The principal purpose was to determine which sensing tasks have been demonstrated, how the evidence was generated, and which industrial requirements remain insufficiently addressed.

The search covered the period from January 2021 to 30 June 2026. Scopus and Web of Science Core Collection were used as the main bibliographic sources. Publisher platforms, DOI records, and Crossref were used to verify bibliographic data. Preprints were excluded if a peer-reviewed version was available, and records with unconfirmed or inconsistent metadata were deleted. The methodological standards and contextual publications cited to position the proposed methodology or to discuss the results after the completion of the systematic search were not retrospectively included in the corpus of 98 technical studies and did not participate in the SRI assessment unless they met the pre-established inclusion criteria.

### 3.2. Search Strategy and Eligibility Criteria

The search combined flotation terms with sensing, machine vision, soft sensors, temporal modelling, uncertainty, and industrial integration. Table 2 summarizes the search groups and the eligibility criteria applied during study selection.

Figure 4 summarizes the PRISMA-based selection workflow. Of 286 identified records, 225 proceeded to title and abstract screening, 152 full texts were assessed, and 98 peer-reviewed technical publications were retained for qualitative synthesis; two additional PRISMA sources support the reporting methodology.

### 3.3. Data Extraction and Coding

For each publication, bibliographic data, the type of ore or material, the flotation stage, laboratory/pilot/industrial scale, data source, camera configuration, data collection duration, target variable, algorithm family, temporal representation, validation scheme, published metrics, uncertainty accounting, inference latency, and automation interface were encoded. This subsection defines only the fields extracted for subsequent validation analysis; the critical interpretation of the strength of validation evidence is concentrated in Section 4.8. Review papers were used to identify field-level trends but were not assigned predictive performance scores.

Table 3 defines the evidence extracted for six appraisal dimensions: measurement definition, acquisition reproducibility, temporal integrity, model validity, operational robustness, and automation integration.

### 3.4. Sensor Readiness Index

The five-dimensional Sensor Readiness Index (SRI) was introduced to summarize evidence of readiness for implementation. Each dimension is rated from 0 to 2: 0—not reported; 1—partially considered; 2—explicitly presented and confirmed.

From the perspective of maturity assessment, SRI operates at a different analytical level than Technology Readiness Level [101]. TRL provides a system-independent assessment of technological maturity; however, a high TRL for a camera, analyzer, computing platform, or communication component does not in itself confirm that the assessment generated by the model is a reliable technological measurement. Sensor readiness additionally requires evidence that the measured quantity is defined and reproducible, the input data and labels are temporally consistent, the validation corresponds to the intended deployment area, degraded and non-distributive states are identified, and the assessment is transmitted to the higher-level system with an explicit indication of validity. For this reason, SRI has been developed as a specialized evidence-based model for intelligent sensors, complementing the overall assessment of technological maturity rather than replacing it. The index for study i is (5):

(5) $${SRI}_{i}=\frac{m_{i}+t_{i}+v_{i}+r_{i}+g_{i}}{10},\;\;0\leq {SRI}_{i}\leq 1,$$

where $m_{i}$ denotes metrological validity, $t_{i}$ temporal integrity, $v_{i}$ validation rigor, $r_{i}$ operational robustness, and $g_{i}$ automation integration. The SRI is not a certification metric and does not replace task-specific performance indicators. Its purpose is to make missing evidence visible and to prevent an offline model with high accuracy from being interpreted automatically as an industrial sensor.

Table 4 presents the five Sensor Readiness Index dimensions and the scoring rules used to distinguish unreported, partially addressed, and explicitly validated evidence.

### 3.5. Methodological Appraisal

Formal risk-of-bias instruments developed for clinical intervention studies (e.g., Cochrane RoB 2, ROBINS-I) are not applicable to the engineering and technical literature synthesized in this review, which does not report patient-level outcomes or randomized/non-randomized treatment comparisons. Instead, methodological quality and evidentiary strength were appraised using the data-extraction and critical-appraisal framework in Table 3 and the five-dimensional Sensor Readiness Index described above, which jointly evaluate measurement definition, acquisition reproducibility, temporal integrity, validation rigor, operational robustness, and automation integration for each included study. Studies with unclear or unreported evidence on a given dimension were scored accordingly (0, not reported) rather than excluded, so that gaps in the evidence base remain visible in the synthesis.

## 4. Results and Critical Synthesis

To directly compare the synthesis results with research questions while maintaining the logic “measurement → modeling → validation → implementation”, the evidence base is structured as follows: RQ1 is disclosed mainly in Section 2.1, Section 2.2, Section 2.3 and Section 2.4 and Section 4.1, Section 4.2 and Section 4.3; RQ2 in Section 4.4, Section 4.5, Section 4.6 and Section 4.7 and Section 4.10; RQ3 in Section 2.2, Section 4.6, Section 4.8 and Section 4.12; RQ4 in Section 3.4 and Section 4.8, Section 4.9, Section 4.10, Section 4.11, Section 4.12 and Section 4.13; RQ5 in Section 5. Several subsections simultaneously relate to more than one research issue, since the tasks of data acquisition, synchronization, validation and assessment of readiness for implementation are interrelated.

### 4.1. Structure of the Recent Evidence Base

The recent evidence base is dominated by machine vision and data-driven state estimation. The strongest publication concentration concerns froth-image segmentation, bubble characterization, condition recognition, and grade or ash-content prediction. A smaller but growing group of studies addresses multimodal fusion, digital twins, physics-informed modelling, and transfer across operating stages. The balance of publications indicates that the research problem has shifted from proving that froth images contain useful information to determining how reliably that information can be converted into a maintained process signal.

Three technological transitions are evident. First, handcrafted colour, texture, and morphology descriptors are increasingly supplemented or replaced by learned features. Second, static-frame classification is moving toward video sequences, recurrent networks, transformers, graph models, and self-supervised learning. Third, model outputs are shifting from descriptive image classes toward technologically meaningful variables such as grade, ash content, froth velocity, operating-performance class, and near-future process behavior.

The evidence remains uneven. Many studies describe neural-network architecture in detail but provide limited information about camera lens, field of view, mounting rigidity, illumination spectrum, exposure policy, optical-window maintenance, or time synchronization. Dataset duration and campaign structure are frequently omitted. Industrial datasets are commonly proprietary and collected from one ore type, cell, or operating period, which restricts independent replication and makes transferability difficult to assess.

Engineering consequence. The strength of the evidence should be assessed independently of the novelty of the model architecture: a comparison of studies on flotation sensing should take into account the data acquisition conditions, campaign structure, temporal grouping, and implementation conditions, rather than ranking models based solely on a single final metric.

To enhance the transparency of the evidence base and facilitate navigation through the literature, the publications in Table 5 are grouped according to their main role in this review and by year of publication. This presentation does not replace a thematic synthesis, since individual studies simultaneously belong to several areas, but it allows one to trace the development of the main research lines over the period 2021–2026.

### 4.2. Image Acquisition as a Metrological Stage

A flotation camera is often described as a convenient source of images, whereas an industrial visual sensor requires a defined measurement geometry. The region of interest must remain fixed relative to the froth surface or be corrected using a geometric reference. Pixel dimensions must be related to physical dimensions when bubble size or velocity is reported. Exposure, gain, white balance, frame rate, illumination position, polarization, and compression influence colour, texture, glare, and apparent bubble boundaries.

**Figure 5:**
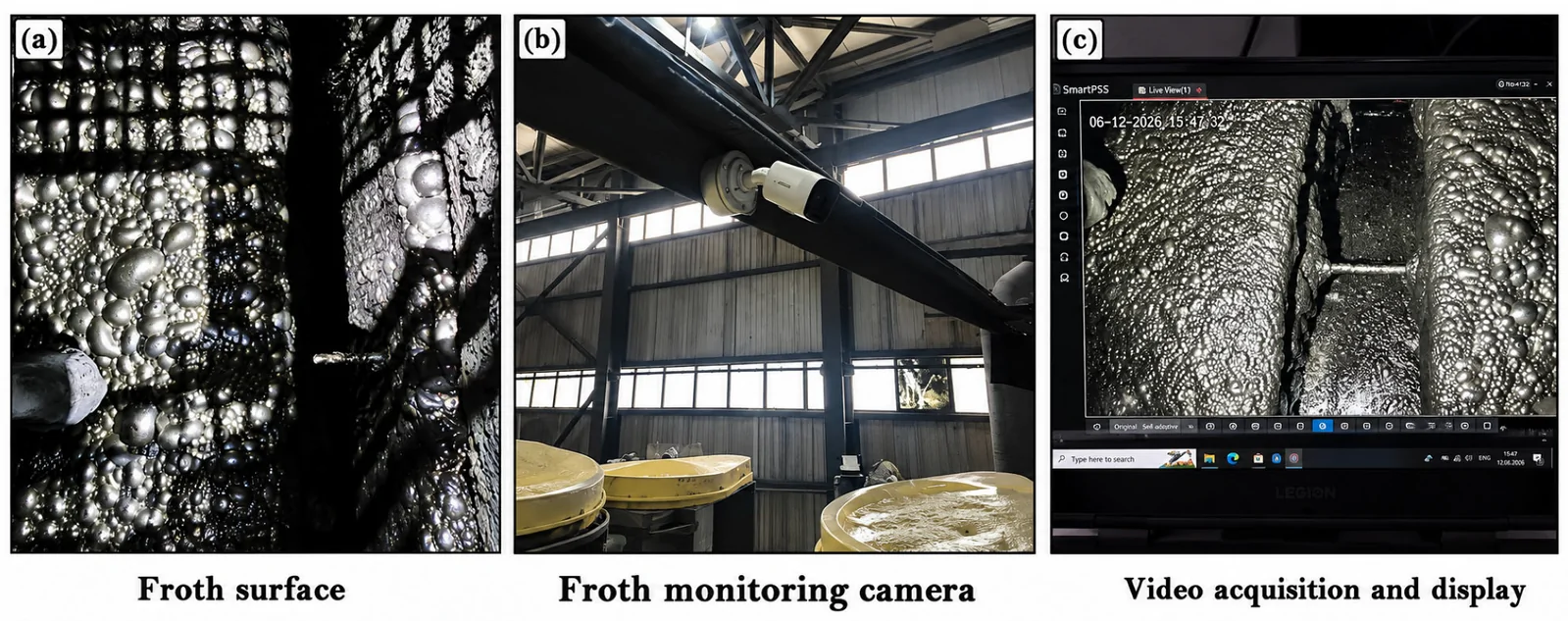
Industrial context of the machine vision system: (**a**) foam surface with local highlights; (**b**) protected camera installed above the flotation machine; (**c**) video capture system and operator display (photos by the authors).

Figure 5 shows a representative industrial machine-vision installation, including froth-surface glare, a protected camera mounted above the cell, and the operator-side video display. These elements illustrate the difference between laboratory imagery and full-scale measurement conditions.

**Figure 6:**
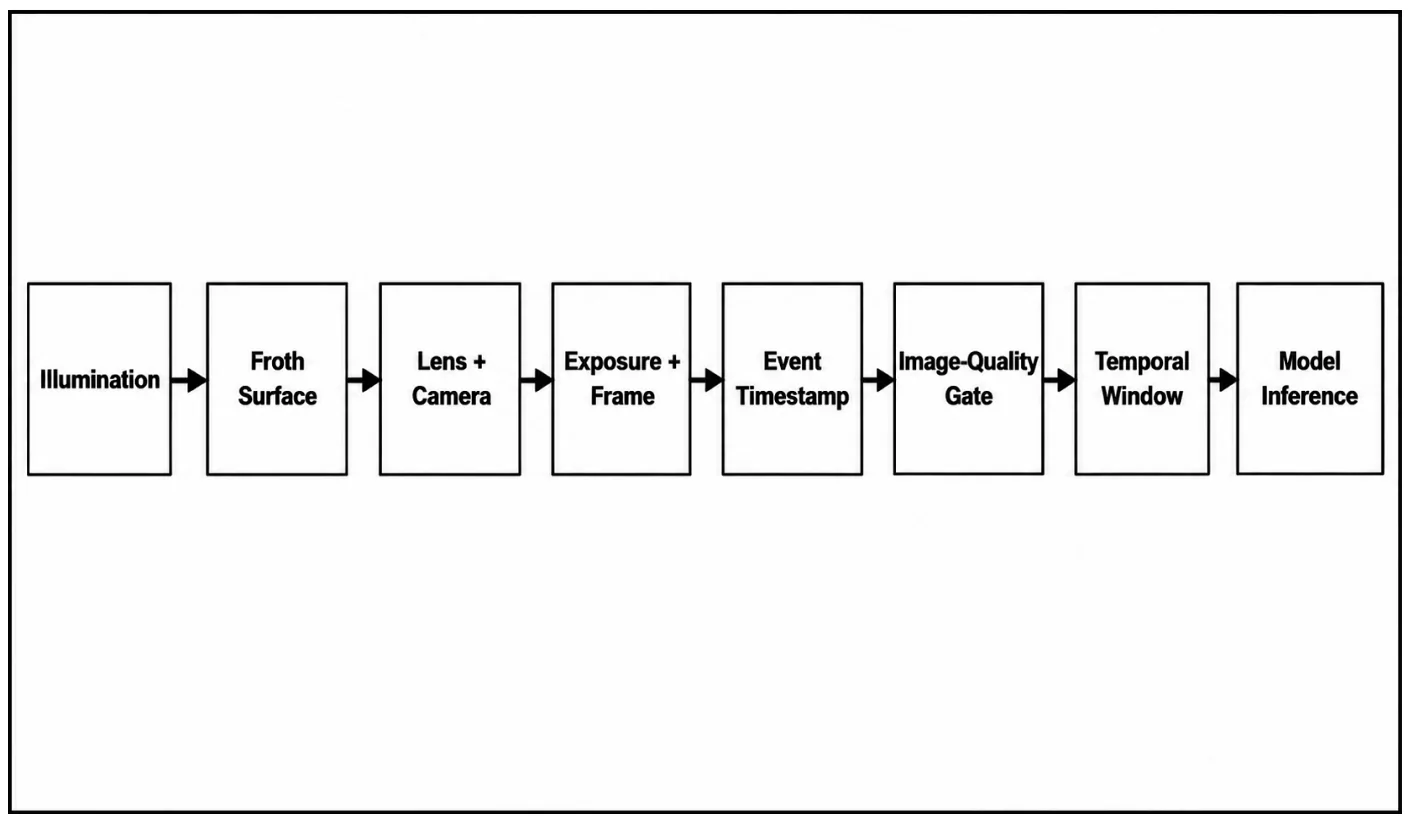
Functional workflow of the visible-spectrum froth-surface image acquisition channel and its associated temporal metadata.

For the industrial visible spectrum channel, image acquisition should be considered as a traceable measurement sequence, not as the acquisition of a single frame. Figure 6 separates the lighting and the fixed field of view of the foam surface, optical image formation, exposure and frame acquisition, event timestamp, image quality control, time window formation, and model inference. Spatial resolution should be characterized at two levels: the camera’s inherent resolution in pixels and, if calibration is available, the physical scale of the image, for example, mm/pixel. The frame acquisition period should be linked to the process dynamics via Δ*tcam* = 1/*fcam*, the exposure time *texp*, and the duration of the time window *Tw* used by the model. Since the studies under consideration use heterogeneous cameras and technological channels, this review does not specify a single universal spatial resolution or acquisition period. Instead, the inherent resolution, physical scale, frame rate/acquisition period, exposure mode, and duration of the time window are considered as mandatory parameters for presenting the results, provided they are published in the original study.

The diagram reflects the sequential transformation of visual information from acquisition of the froth-surface image to the formation of a verified model estimate of the flotation process state.

Physical installation determines the lower bound of achievable uncertainty. A network cannot recover information that is absent because bubbles are below the effective spatial resolution, the surface is saturated by glare, the optical window is contaminated, or the field of view has shifted. Image enhancement can improve visibility, but it should not be used to conceal unstable acquisition. A maintained visual channel should therefore include a commissioning record, camera coordinates, lens and working distance, spatial scale, illumination configuration, reference images, cleaning interval, and acceptance limits for sharpness and exposure.

A minimum image-quality vector may be defined as (6):

(6) $$q_{k}^{img}={\left[q_{sharp},q_{exp},q_{glare},q_{occ},q_{geom},q_{age}\right]}_{k},$$

where the components describe sharpness, exposure, glare fraction, occlusion or contamination, geometric consistency, and frame age. The model should either receive these indicators as inputs or be prevented from generating an operational estimate when the quality vector falls outside validated limits.

Engineering consequence. The camera installation must be commissioned and maintained as a metrological subsystem; model development must not be carried out independently of checking the field of view, physical scale, lighting stability, and the condition of the protective optical window.

### 4.3. Preprocessing and Image-Quality Control

Preprocessing in flotation commonly includes region-of-interest selection, colour normalization, denoising, illumination correction, contrast enhancement, resizing, and frame selection. These operations are often presented as implementation details, but they define the measurement transfer function. An enhancement method may alter bubble boundaries or texture statistics, while aggressive resizing may remove small bubbles. Accordingly, preprocessing parameters should be versioned and validated together with the model.

Data augmentation can improve robustness to moderate changes in brightness, contrast, orientation, or scale, but unrealistic transformations may break the relationship between visual appearance and process state. Vertical flips, strong hue shifts, or geometric distortions can produce images that are statistically diverse but physically implausible. Augmentation should therefore be constrained by the disturbances expected in the target installation.

Image-quality control should precede process inference. A two-stage design is preferable: the first stage determines whether the image belongs to the validated acquisition domain; the second estimates the process variable. This structure allows the system to distinguish “poor image quality” from “abnormal flotation state”. Without such separation, a contaminated lens may be interpreted as a process disturbance.

Engineering implication. The parameters of preprocessing and image quality control must be versioned together with the model, and a frame that does not meet the quality requirements must be rejected before performing the technological inference, rather than being converted into a plausible but unreliable assessment of the process.

### 4.4. Bubble Segmentation and Physical Descriptors

Bubble-size distribution, froth velocity, colour, texture, stability, and mobility remain central because they are interpretable and can be related to hydrodynamics and mineral transport. Recent methods include open-source bubble analysis, instance segmentation, U-Net-based semantic segmentation, fast bubble-size measurement, and deep learning for joint size and velocity estimation. These approaches reduce sensitivity to overlapping bubbles and irregular boundaries compared with classical thresholding and watershed methods, but their metrological validity depends on annotation quality and spatial calibration.

A bubble-size distribution should not be reduced automatically to one mean value. A robust visual sensor should retain quantiles, variance, skewness, local spatial distribution, and the fraction of unresolved or unsegmented area. If $d_{n}$ denotes the equivalent diameter of a segmented bubble n, a compact descriptor is (7):

(7) $$b_{k}={\left[d_{10},d_{50},d_{90},\bar{d},sd\left(d\right),\gamma_{d},\rho_{unseg}\right]}_{k},$$

where $\gamma_{d}$ is distribution skewness and $\rho_{unseg}$ is the unsegmented-area fraction. Reporting $\rho_{unseg}$ prevents an apparently precise distribution from being accepted when a substantial portion of the image cannot be resolved.

Velocity estimation has evolved from block matching and optical flow toward convolutional feature matching, semantic segmentation, recurrent forecasting, and graph representations of bubble trajectories. These methods are especially relevant because motion provides information about mass transport and impending changes in overflow. Nevertheless, velocity estimates require camera-motion compensation and a physical spatial scale. A network can produce numerically stable pixel displacement while the camera itself is vibrating.

Engineering implication. Estimations of bubble sizes and foam velocity require verification of the physical scale, control of camera movement, and an explicit indicator of the fraction of the unresolved area; high precision at the pixel level alone is insufficient to form a quantitative industrial sensor.

### 4.5. State Recognition and Operating-Performance Assessment

Operating-condition recognition converts visual observations into discrete states such as stable operation, excessive aeration, low reagent dosage, unstable froth, or abnormal mobility. Convolutional networks remain common because they learn spatial features directly from images. Transformer and attention-based models extend the receptive field and can represent long-range structure. Ensemble sensors reduce model variance, while explainability methods can reveal whether predictions depend on physically meaningful regions or on irrelevant background cues.

The principal validation risk is temporal leakage. Consecutive video frames are strongly correlated. Random frame-level splitting allows nearly identical observations from the same operating interval to appear in training and test sets, producing optimistic accuracy. A valid evaluation should split by campaign, operating interval, shift, cell, or ore domain before individual frames are sampled. For a video dataset containing campaigns $C_{1},\ldots ,C_{m}$, the training and test sets should satisfy (8):

(8) $$C_{train}\cap C_{test}=⌀,$$

rather than merely ensuring that individual image files are different.

Operating-performance assessment is more demanding than image classification because the state label must be tied to a process criterion. A visual class defined by operator judgment may be useful, but its reproducibility and relationship to grade, recovery, or stability should be demonstrated. Multi-rater annotation, inter-rater agreement, and explicit label definitions strengthen the evidence.

Engineering consequence. The validation unit for state recognition from video should be a technological campaign, a work interval, a shift, a flotation machine, or an area of ore raw material, rather than an individual frame; this prevents temporary data leakage and artificial overestimation of accuracy.

### 4.6. Temporal Models and Future-State Prediction

Temporal data alignment and label generation are carried out in accordance with the approach defined in Section 2.2; therefore, this subsection focuses on dynamic input windows, forecasting horizons, and the possibility of industrial application of temporal models. Froth behavior is dynamic. Coalescence, drainage, transport, and collapse develop over time, and the same static appearance may correspond to different trajectories. To account for the dynamics of the process, content monitoring models based on LSTM [21], encoder–decoder models for time-series forecasting [22], industrial ConvLSTM monitoring models [34], LSTM forecasting based on image sequences [44], graph representations of foam velocity [46], and transformer models for time-based feature fusion [64,65] are used. These approaches process image sequences or multimodal time windows and are applied for content monitoring, tail forecasting, foam velocity estimation, and process state recognition.

The input horizon and prediction horizon should be selected from process dynamics rather than only by hyperparameter search. A very short window may not capture relevant transport behavior, while a long window increases latency and may average across regime changes. For a model with input horizon L and prediction horizon h, the operational value depends on whether (9):

(9) $$\tau_{acq}+\tau_{window}+\tau_{prep}+\tau_{inf}+\tau_{comm}<h\Delta t,$$

where the terms represent acquisition, window formation, preprocessing, inference, and communication latency. A prediction that becomes available after the predicted event has already occurred has little supervisory value, even if its offline error is low.

Future-state models should be evaluated recursively or at all forecast horizons. Reporting only the first step hides error accumulation. Prediction intervals should widen with the horizon, and the system should distinguish between interpolation within a known regime and extrapolation after an ore or equipment change.

Engineering consequence. The temporal model has practical value only if its confirmed forecast horizon exceeds the total delay from data receipt to decision-making and remains stable when the technological mode changes.

### 4.7. Visual and Multimodal Soft Sensors

Visual soft sensors estimate variables that are difficult or delayed to measure directly, including concentrate grade, tailings grade, sulfur removal, silica impurity, recovery, and ash content. Recent studies use handcrafted features, pretrained CNNs, hybrid feature extraction, Raman spectroscopy, semi-supervised learning, transfer learning, physics-informed models, and multimodal transformers. These approaches demonstrate that visual and process data provide complementary information.

For the purposes of this synthesis, the multimodal strategies presented in the reviewed literature can be grouped into early, intermediate, and late data fusion [37,63,64]. Early fusion involves concatenating synchronized source or low-level features; intermediate fusion involves integrating hidden representations of individual modalities; late fusion involves combining independently generated predictions or decisions. Representative implementations in flotation include the multimodal temporal hypergraph model [37], the combination of foam and tail images based on a stacking approach [63], and the transformer for intertemporal feature fusion [64]. Early fusion is simple but sensitive to missing channels and scale differences; intermediate fusion is more flexible but less interpretable; late fusion facilitates modular diagnostics, but may not fully account for intermodal interactions.

A quality-aware multimodal estimate can be written as (10):

(10) $${\hat{y}}_{k}=\sum_{m=1}^{M}w_{m,k}{\hat{y}}_{m,k},\;\;w_{m,k}=\frac{exp\left(g_{m}\left(q_{m,k}\right)\right)}{\sum_{r=1}^{M}exp\left(g_{r}\left(q_{r,k}\right)\right)},$$

where ${\hat{y}}_{m,k}$ is the estimate from modality m and the weight $w_{m,k}$ depends on the modality-quality vector. This structure illustrates a practical principle: the model should rely less on a channel when its quality deteriorates.

Spectroscopic and analytical modalities are particularly valuable for calibration because they measure composition more directly than images. Raman-based concentrate-grade prediction demonstrates the potential of in situ spectroscopy, while image–process fusion improves continuity when analyzer measurements are sparse. However, analyzer data should not be treated as error-free ground truth. Sampling representativeness, calibration uncertainty, and delay must be included in the label model.

Engineering consequence. Multimodal models should be tested in the event of the absence, obsolescence, or degradation of individual channels; instead of unconditional feature combination, quality-dependent channel weighting or an explicitly defined fallback strategy should be used.

### 4.8. Validation, Uncertainty, and Domain Shift

Taking into account the temporary coordination scheme defined in Section 2.2, validation is assessed here in relation to the intended area of implementation. Four levels of evidence are distinguished: random internal sampling; chronological sampling; inter-company or inter-machine validation; external validation at another enterprise or ore type. The first level measures interpolation under nearly identical conditions. The fourth provides the strongest evidence of transferability.

Uncertainty is required for operational interpretation. Deep ensembles, Bayesian approximations, conformal methods, quantile regression, and probabilistic forecasting can provide intervals or distributions. Regardless of method, uncertainty should be calibrated. If a nominal (1−α) prediction interval is used, empirical coverage should satisfy (11):

(11) $$PICP=\frac{N_{covered}}{N_{total}}\approx 1-\alpha .$$

Poorly calibrated confidence scores may be more dangerous than no uncertainty information because they create unjustified operator trust.

Domain shift arises from ore mineralogy, particle size, reagent suite, camera replacement, lighting, seasonal conditions, cell geometry, and equipment maintenance. Transfer learning can reduce retraining requirements, but its success should be quantified separately for representation transfer and output calibration. A model that retains classification accuracy but produces biased grade estimates still requires recalibration.

Out-of-distribution detection should be connected to a defined fallback action. When the input falls outside the validated domain, the system may hold the last valid estimate for a limited period, switch to a simpler process-data model, revert to advisory mode, or suppress the output. The fallback policy should be tested with simulated and real faults.

Engineering consequence. Uncertainty assessment and identification of data outside the training distribution acquire industrial relevance only when they are associated with clear indicators of signal validity and verified backup actions of the system.

### 4.9. Industrial Integration and End-to-End Latency

**Figure 7:**
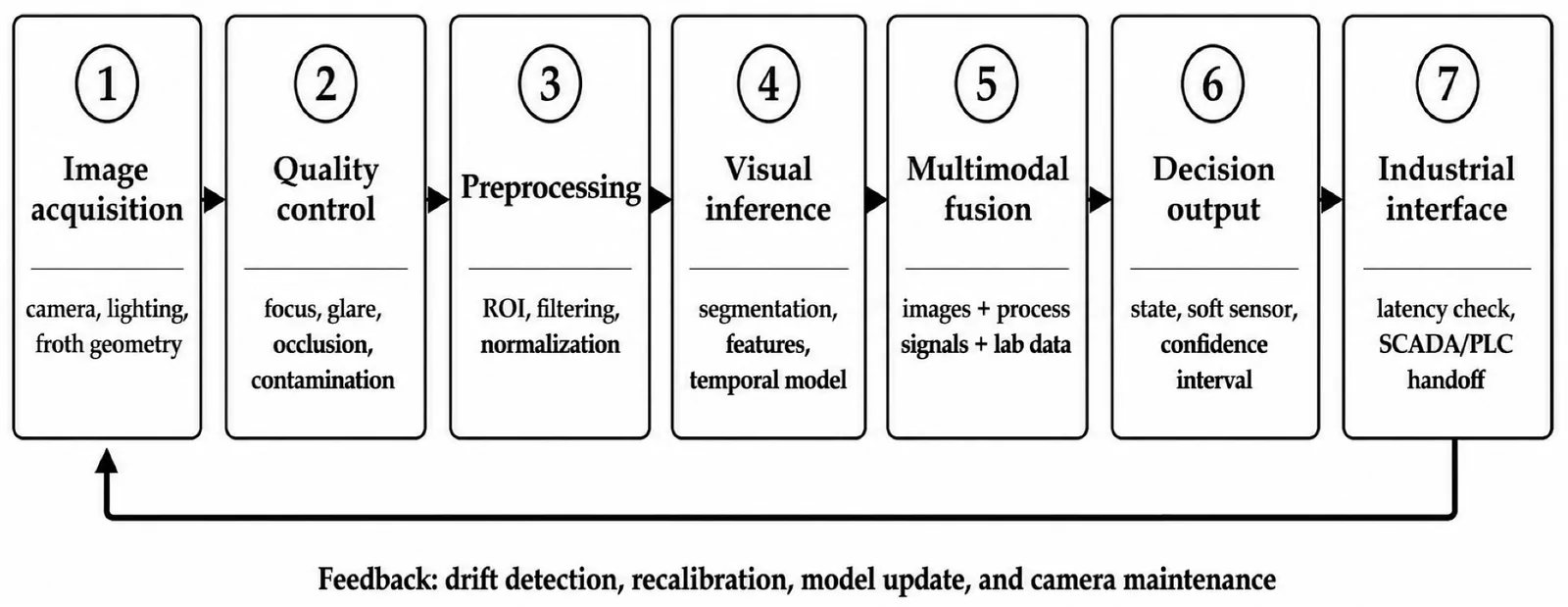
Functional workflow of a multimodal flotation sensor system. The diagram separates data acquisition, synchronization, quality control, state estimation, industrial communication, and feedback. The archived inference record includes the event timestamp, channel quality status, model version, prediction uncertainty, validity status, and automation interface state.

Figure 7 presents the end-to-end multimodal sensing-system workflow: from data acquisition and synchronization through quality control and state estimation to industrial communication, archival traceability, and feedback for drift detection and maintenance.

The total latency is (12):

(12) $$\tau_{\Sigma }=\tau_{acq}+\tau_{sync}+\tau_{prep}+\tau_{inf}+\tau_{comm}+\tau_{PLC}+\tau_{disp},$$

where acquisition, synchronization, preprocessing, inference, communication, controller processing, and display or actuation delays are included. Reporting only neural-network inference time is insufficient. A model that requires a 60 s aggregation window and 2 s data transfer cannot be described as a 20 ms sensor merely because the forward pass takes 20 ms.

The historian should archive raw measurements, processed features, prediction, uncertainty, validity flag, model version, operator mode, AI recommendation, PLC command, actual actuator position, and interlock status. This record allows errors to be separated into measurement error, model error, communication failure, actuator limitation, or operator override.

Engineering consequence. Full delay, model version, input data quality, forecast, operator action, PLC command, and the actual response of the actuator must be archived as a single traceable inference record.

### 4.10. Comparative Synthesis by Sensing Task

Table 6 compares the dominant flotation sensing tasks, the metrics most appropriate for each task, and the validation limitations that prevent ranking studies by a single accuracy value.

### 4.11. Sensor Readiness Index Synthesis

Application of the SRI framework indicates that the strongest evidence in the recent literature concerns predictive validity under internal datasets. Metrological validity is moderate where camera setup and image resolution are reported, but detailed illumination, cleaning, and geometric verification remain uncommon. Temporal integrity is highly variable; sequence models often describe input windows but do not always document the physical relation between video and assay labels. External validation and calibrated uncertainty are rare. Automation integration is usually limited to conceptual architectures or real-time prototypes without complete end-to-end latency and fallback testing.

To directly verify the central thesis, Table 7 compares representative studies demonstrating high predictive or online performance with the actual evidence presented regarding methodological validity, temporal integrity, validation rigor, operational stability, and integration with automation. The table is illustrative rather than rating-based, since SRI reflects the completeness of published evidence of readiness rather than the absolute superiority of the algorithm.

The comparison shows that a very high test score may be combined with weak evidence of campaign independence, uncertainty, failure behavior, or transfer to the automation system. In contrast, pilot studies that explicitly test mode changes, sampling frequency, uncertainty, and online adaptation may have stronger evidence of readiness, even without a single impressive final accuracy metric. Therefore, predictive indicators and evidence of readiness should be presented together and should not be used as interchangeable characteristics.

The SRI should therefore be interpreted as a profile rather than only a scalar value. Two models may have the same total score while differing substantially: one may have excellent acquisition quality but weak external validation; another may have strong predictive validation but no automation interface. A radar profile or dimension-wise table is more informative for engineering decisions than a single ranking.

### 4.12. Cross-Study Evidence Patterns and Limits of Comparability

The reviewed publications demonstrate clear technical progress, but direct ranking of algorithms remains methodologically unsafe. Reported metrics are conditioned by ore type, flotation stage, image field of view, camera resolution, label definition, operating variability, and data-partition strategy. A lower RMSE obtained for one concentrate-grade dataset cannot be interpreted as evidence of a universally superior sensor when another study uses a different sampling delay, target range, assay frequency, or campaign structure. The same limitation applies to segmentation scores: intersection-over-union is affected by annotation policy, treatment of unresolved bubbles, image scale, and the definition of touching boundaries. Cross-study synthesis should therefore compare evidence classes and validation designs rather than only numerical scores.

Four evidence levels can be distinguished. The first level establishes algorithmic feasibility using a curated image or process dataset. These studies are valuable for testing architectures and identifying informative features, but they provide limited evidence concerning installation stability or temporal transfer. The second level evaluates a model on chronological or campaign-separated data from one plant. This design addresses temporal leakage and begins to reveal concept drift, although transfer to another cell or ore domain remains unknown. The third level includes inter-cell, inter-campaign, or external validation and therefore provides stronger evidence of generalization. The fourth level demonstrates a maintained online signal with documented latency, quality flags, uncertainty behavior, operator visibility, and fallback logic. Most recent studies occupy the first two levels; comparatively few reach the third level, and complete fourth-level evidence remains uncommon [16,17,18,19,20,21,22,23,24,25,26,27,28,29,30,31,32,33,34,35,36,37,38,39,40,41,42,43,44,45,46,47,48,49,50,51,52,53,54,55,56,57,58,59,60,61,62,63,64,65,66,67,68,69,70,71,72,73,74,75,76,77,78,79,80,81,82,83,84].

The evidence also differs by sensing task. Bubble segmentation and froth velocity estimation have relatively clear physical targets, but their accuracy depends on scale calibration and reference measurements. State recognition is easier to formulate computationally, yet the process meaning of the classes may be subjective unless the labels are linked to operational criteria. Grade and ash-content soft sensors have direct metallurgical relevance, but they depend on delayed and frequently sparse laboratory labels. Multimodal models can improve prediction by combining images and process tags, although they introduce additional synchronization and missing-channel risks. These differences explain why one universal metric is unsuitable for the field.

The reviewed studies further indicate that model complexity and evidence strength are only weakly related. Transformer, graph, ensemble, and self-supervised architectures can provide useful representations, especially when long-range spatial or temporal dependencies are relevant [24,25,26,27,28,29,30,31,32,33,34,35,36,37,38,39,40,41,42,43,44,45,46,47,48,49,85,86,87,88,89,90,91,92,93,94,95,96,97,98,99,100]. Nevertheless, a simpler model evaluated on a long industrial campaign with a strict chronological split may provide more credible deployment evidence than a more complex network assessed through random frame splitting. Architectural novelty should be treated as one contribution dimension, whereas measurement definition, dataset construction, and validation rigor should be treated as independent dimensions.

The comparability between studies is further limited if the visual, technological, and laboratory target variables have different spatial or temporal support. The rules for physically consistent delay compensation and tag aggregation are defined in Section 2.2 and are not repeated here.

Two additional process-oriented studies on flotation, cited outside the PRISMA framework, further illustrate the difference between evidence of the technological efficiency of flotation and evidence of the sensor’s readiness. Cheng et al. [102] reported achieving a combustible mass recovery rate of up to 85.23% by intensifying the flotation of fine-dispersed slag from coal gasification, whereas Cheng et al. [103] obtained an 88.49% combustible mass recovery rate using CO_2_-mediated flotation of low-grade coal and a 17–22% reduction in the global warming indicator compared to conventional air flotation. These results provide strong evidence of the effectiveness of the flotation process itself, but they do not in themselves confirm the metrological reproducibility, temporal integrity, external validation of the sensor, uncertainty quantification, or integration with the automation system. Therefore, these works are used as contextual examples and are not included in the SRI synthesis of the systematic corpus.

The same distinction applies to uncertainty. Predictive dispersion can originate from measurement noise, limited training coverage, process non-stationarity, ambiguous labels, or a degraded acquisition channel. A single confidence interval does not identify the source. For industrial use, uncertainty should be decomposed where possible into measurement-quality uncertainty and model uncertainty. This allows a contaminated optical window, an out-of-domain ore condition, and an intrinsically variable process state to generate different maintenance or operating responses. The literature increasingly recognizes uncertainty and out-of-distribution detection, but calibration under realistic process disturbances remains limited [89,90,91,92,93,94,95,96,97,98,99,100].

The comparative synthesis supports an evidence hierarchy for future review and experimental work. Primary importance should be assigned to reproducibility of acquisition and target construction. Secondary importance should be assigned to chronological and external validation, including confidence intervals and baseline comparisons. Operational robustness should then be assessed through controlled perturbations or naturally occurring disturbances, such as lighting change, lens contamination, camera displacement, missing process tags, analyzer delay, and ore-domain change. Only after these stages should real-time performance and automation integration be interpreted. This hierarchy prevents a high offline score from being presented as equivalent to an industrial measurement channel.

### 4.13. Reference Architecture for an Automation-Ready Flotation Sensor

The synthesis permits a more explicit reference architecture to be formulated. The first layer is the physical acquisition layer, comprising field instruments, cameras, online analyzers, laboratory sampling, and equipment feedback. Each channel should generate not only a value but also acquisition time, source identifier, engineering units, quality state, and calibration or maintenance status. The second layer is temporal harmonization, where transport delays, resampling policies, composite-sample intervals, and video-window definitions are applied. The third layer performs quality assessment and feature extraction. Invalid channels are masked or routed to a defined fallback rather than silently imputed without traceability.

The fourth layer is the state-estimation model. Its output should contain the predicted variable, uncertainty or confidence information, the valid operating domain, and the model version. The fifth layer is an independent supervisory gate. This gate checks data quality, uncertainty, model age, inference latency, and process constraints before the estimate is exposed to the operator or an advanced-control application. The sixth layer is the historian and governance layer, which archives the complete inference record and supports audit, drift analysis, retraining, and rollback. Separation of the estimation model from the supervisory gate is essential because statistical confidence and process permissibility are not identical.

For advisory operation, the minimum output package is a timestamped estimate, confidence or validity indicator, current image or channel quality, and an explanation of why an estimate is unavailable. For closed-loop use, additional evidence is required: deterministic communication with the PLC or supervisory controller, verified end-to-end latency, handling of stale values, interlock behavior, rate and saturation limits, operator override, and a safe fallback state. The sensing article should report these interfaces even when control performance is outside its scope, because the usability of a sensor depends on the downstream decision cycle [71,72,73,74,75,76,77,78,79,80,81,82,83,84].

Acceptance testing should be based on scenarios rather than a single aggregate metric. Normal-operation tests verify bias, precision, repeatability, and latency. Transition tests verify response during changes in feed, aeration, level, or reagent dosage. Degradation tests introduce controlled changes in illumination, optical contamination, missing tags, and delayed labels. Domain tests evaluate new campaigns, ore blends, or cells. Recovery tests verify that the system returns to a valid state after maintenance or communication loss. For every scenario, the expected model output, quality flag, uncertainty behavior, and fallback response should be specified in advance.

The proposed architecture also clarifies the boundary between sensing and control. A data-driven model becomes a sensor when it produces a defined and quality-controlled estimate of process state. It becomes part of an advanced-control system only when that estimate is consumed by a decision layer with explicit constraints and feedback verification. Keeping these roles distinct allows a review article to remain self-contained while still establishing the engineering requirements for subsequent optimization or control studies.

## 5. Research Gaps and Deployment Framework (RQ5)

### 5.1. Reproducible Visual Measurement

The first research gap is the absence of standardized acquisition reporting. Future studies should provide the camera model, lens, working distance, field of view, image resolution, frame rate, exposure and gain policy, illumination geometry, polarization, mounting method, optical protection, cleaning interval, spatial calibration, and representative raw images. Reporting only network input size is not sufficient because resizing does not describe the original measurement resolution.

A calibration object or stable geometric feature should be used to verify camera displacement. Where direct calibration above the operating cell is impractical, commissioning should establish a reference field of view and an automated registration test. Colour-based features require a white-balance and illumination policy. Texture-based features require compression and sharpening settings to remain fixed.

### 5.2. Benchmark Datasets with Campaign Structure

Public datasets have improved reproducibility, but most available collections do not represent long-term industrial disturbances. A useful benchmark should contain multiple campaigns, cells, ore domains, illumination conditions, maintenance events, and process transitions. Metadata should include timestamps, process tags, analyzer and laboratory labels, image-quality indicators, and explicit grouping variables for leakage-free validation.

Benchmark design should distinguish three tasks: within-campaign interpolation, cross-campaign generalization, and cross-site transfer. A leaderboard based only on randomly shuffled frames would reinforce the existing validation problem. Performance should be reported for each domain and with uncertainty.

### 5.3. Uncertainty-Qualified Sensing

A process estimate should be accompanied by uncertainty and validity information. Future research should compare probabilistic approaches not only by interval width but also by calibration, sharpness, and behavior under shift. Uncertainty should increase when image quality deteriorates, a modality disappears, or the process enters an unfamiliar domain. If uncertainty remains artificially low under these conditions, the model is unsuitable for operational use.

The operator interface should distinguish measurement uncertainty from process variability. A wide prediction interval due to poor image quality requires maintenance or fallback; a rapidly changing but precisely measured state requires process attention. These situations should not generate the same alarm.

### 5.4. Continuous Adaptation and Model Governance

Flotation processes change because of ore variability, reagent changes, equipment wear, camera replacement, and operational improvements. Continuous adaptation is therefore necessary, but uncontrolled online learning can introduce silent degradation. Model updates should be governed by version control, validation gates, rollback capability, and a record of the data used for retraining.

A practical lifecycle includes drift detection; collection of representative labels; offline retraining; chronological and domain validation; shadow-mode comparison; controlled activation; and post-deployment monitoring. Automatic adaptation should be limited to parameters with a defined safety envelope unless stronger assurance is available.

### 5.5. Minimum Reporting Checklist

Table 8 consolidates the review findings into a minimum reporting checklist for manuscripts, technical reports, and pilot-scale or industrial validation protocols.

### 5.6. Recommended Industrial Validation Sequence

A staged validation strategy reduces risk. Stage 1 verifies camera and sensor reproducibility under controlled conditions. Stage 2 performs offline chronological validation using historian data. Stage 3 operates the model in shadow mode and compares predictions with assays and operator assessments. Stage 4 provides advisory outputs with quality and uncertainty indicators. Stage 5 permits constrained supervisory use after latency, interlocks, fallback, and operator procedures have been validated.

Closed-loop use should not be claimed when the system only generates recommendations. The executed control action must be archived and the process response evaluated. Similarly, “real time” should be supported by the complete latency budget rather than by inference time alone.

### 5.7. Economic and Deployment Considerations

The readiness of the sensor is a necessary but insufficient condition for industrial implementation. The decision to implement it should be based on the value of the system’s full life cycle, not just on the accuracy of the predictions. The main cost categories include measuring equipment, chambers and protective casings, online analyzers when used, peripheral or server computing, an industrial network, integration with historian/LIMS/SCADA, installation and commissioning, periodic cleaning and calibration, consumables for analyzers, obtaining reference labels, re-validation and retraining of models, cybersecurity, and model version management. The potential effect includes reducing the delay in laboratory analysis, decreasing the overconsumption of reagents, reducing metallurgical losses, reducing the workload on operators, preventing unplanned downtime, and increasing recovery or productivity. Economic feasibility should be assessed separately from the five-dimensional SRI. For each implementation option, it is necessary to specify the main capital and operating costs, the frequency of maintenance, the computing and communication requirements, the expected loss prevention or technological effect, as well as the initial assumptions used to calculate the total cost of ownership, payback period, net present value, and return on investment. A technically mature sensor may remain economically unattractive if the life cycle costs exceed the operational effect, whereas a simpler, more robust, and maintainable architecture may have a stronger industrial justification.

Modern process-oriented studies of flotation show why industrial value should ultimately be expressed through technological and resource indicators, rather than through the accuracy of the model as such. Cheng et al. [102] reported an extraction of combustible mass of up to 85.23% after intensifying the flotation of fine-dispersed slag from coal gasification, and an extraction of 84.10% when using modified methyl oleate at a consumption rate equal to one-third of the diesel reagent consumption. Cheng et al. [103] obtained an 88.49% recovery of combustible material in CO_2_-mediated flotation of low-grade coal, reduced the need for reagents, and reduced the global warming indicator by 17–22% compared to conventional air flotation. These studies do not establish the economics of sensor systems, but they demonstrate the types of effects—recovery, reagent consumption, and environmental load—that the economic justification for sensorics and automation should ultimately be linked to.

Therefore, the economic assessment is considered a parallel criterion for implementation, rather than a sixth dimension of SRI. SRI characterizes the completeness of technical and substantive readiness, whereas the economic assessment determines the justification for implementing a technically viable system for a specific enterprise, ore type, management strategy, and service conditions.

### 5.8. Synthesis of Answers to the Research Questions

RQ1. Flotation state identification is by its nature a multimodal and multi-rate task combining field instruments, machine vision, online and spectroscopic analysis, laboratory reference data and equipment feedback, with each channel having its own time characteristics and sources of uncertainty (Section 2.1, Section 2.2, Section 2.3 and Section 2.4 and Section 4.1, Section 4.2 and Section 4.3).

RQ2. Machine Vision has evolved from manual foam features to convolutional, temporal, transformer, graph, self-supervised, and multimodal models for bubble structure estimation, foam velocity, state recognition, and metallurgical soft sensing (Section 4.4, Section 4.5, Section 4.6 and Section 4.7 and Section 4.10).

RQ3. Reliable synchronization requires event timing matching, explicit delay models, physically consistent aggregation windows, and validation with chronological or campaign separation without data leakage (Section 2.2, Section 4.6 and Section 4.8).

RQ4. A laboratory predictive model becomes an industrial sensor only when the quality of the target assessment is complemented by metrological reproducibility, temporal integrity, validation appropriate to implementation conditions, operational stability, uncertainty and OOD state processing, confirmed delay and traceable automation interfaces (Section 3.4 and Section 4.8, Section 4.9, Section 4.10, Section 4.11, Section 4.12 and Section 4.13).

RQ5. The main unresolved issues remain standardized data acquisition and description, campaign-structured reference sets, external validation, calibrated uncertainty, degradation and channel absence tests, controlled model adaptation, confirmed data transfer to an automated system, and explicit economic assessment of implementation (Section 5.1, Section 5.2, Section 5.3, Section 5.4, Section 5.5, Section 5.6 and Section 5.7).

These brief responses link the research questions to the relevant sections of the evidence synthesis without reiterating the methodological provisions that have already been discussed.

### 5.9. Limitations of the Review

Several limitations should be considered when interpreting the synthesis. First, the review was restricted to English-language peer-reviewed publications issued between 2021 and June 2026. This restriction was adopted to provide a current evidence base, but it excludes earlier foundational studies and may omit relevant regional publications or industrial reports that are not indexed in Scopus or Web of Science. Earlier work was used only indirectly through recent reviews and was not included in the 98-study technical corpus.

Second, quantitative meta-analysis was not appropriate because the reviewed studies use heterogeneous targets, ore types, cameras, sampling intervals, label definitions, dataset partitions, and performance metrics. Reported values of accuracy, RMSE, $R^{2}$, IoU, or velocity error are therefore summarized critically rather than pooled statistically. The absence of a pooled effect size should not be interpreted as a lack of evidence; it reflects insufficient methodological comparability across the field.

Third, many industrial datasets are proprietary and incompletely described. Independent verification of campaign duration, class balance, sampling delays, camera maintenance, and train-test grouping was not always possible. The assessment consequently reflects reported evidence rather than unreported implementation practice. A study may have applied stronger internal procedures than those documented in the publication, but those procedures cannot contribute to reproducible scientific evidence unless they are reported.

Fourth, the proposed Sensor Readiness Index is a review and reporting framework rather than a certified technology-readiness scale. Its dimensions were derived from recurring methodological and engineering requirements identified across the literature. Therefore, SRI should be interpreted as a specialized evidence-assessment model that complements, but does not replace, formal TRL evaluation or certification procedures. Formal validation of inter-rater reliability, weighting, threshold selection, and predictive association with successful deployment remains future work. The dimension-wise profile should therefore be prioritized over the scalar score.

Finally, several 2025–2026 publications represent early-access or recently issued work for which long-term citation impact and independent replication are not yet available. Their inclusion is justified by the rapid development of multimodal, transformer, graph-based, physics-informed, and digital-twin methods, but conclusions concerning industrial maturity remain provisional. These limitations reinforce the need for transparent datasets, explicit temporal grouping, external validation, and complete acquisition and deployment reporting.

## 6. Conclusions

This review’s systematic evidence base on process measurements, machine vision, multimodal soft sensing, and industrial integration for flotation state identification comprises 98 peer-reviewed technical studies from 2021 to June 2026; two PRISMA methodological publications provide the review’s methodology, while additional methodological and contextual sources are cited separately and are not retrospectively added to the systematic evidence corpus. Machine vision is the most mature non-contact sensing approach and has progressed from handcrafted froth descriptors to convolutional, transformer, self-supervised, graph-based, temporal, and multimodal models. Recent studies demonstrate measurement of bubble-size distributions and froth velocity, recognition of operating conditions, prediction of grade and ash content, and integration with process data and digital-twin architectures.

The central finding is that predictive accuracy and sensor readiness are not equivalent. Industrial readiness requires a reproducible measurement geometry, controlled or characterized illumination, image-quality diagnostics, physical time alignment, leakage-free validation, uncertainty calibration, robustness to domain shift, verified end-to-end latency, and an explicit interface with SCADA, historian, PLC, and operator workflows. These requirements are represented by the proposed Sensor Readiness Index, which separates metrological validity, temporal integrity, validation rigor, operational robustness, and automation integration.

The principal research priority is no longer simply the development of more complex neural architectures. Greater value will be obtained from long-duration industrial datasets with campaign structure, multimodal quality indicators, external validation, calibrated uncertainty, missing-channel tests, controlled adaptation, and traceable deployment. A model should be regarded as an industrial flotation sensor only when it produces a defined, timestamped, uncertainty-qualified, and quality-controlled estimate within the required process cycle and fails safely outside its validated operating domain.

Future industrial validation should present not only evidence of sensor-system readiness but also life-cycle costs and the value-justification assumptions required to confirm deployment feasibility.

## Figures and Tables

**Table 1:** Principal measurement channels used in flotation state identification.

Channel Class	Typical Variables or Outputs	Temporal Characteristics	Main Uncertainty and Failure Modes	Role in State Identification
Field instrumentation	Pulp level, airflow, pressure, density, pH, Eh, conductivity, temperature, motor power	Sub-second to several seconds	Drift, fouling, calibration loss, scale mismatch, signal dropout	Fast description of hydrodynamic and chemical state; regulatory context
Machine vision	Bubble-size distribution, velocity, colour, texture, mobility, stability, froth depth, state class	Frames or video windows; inference-dependent latency	Glare, vibration, optical contamination, camera displacement, illumination change, domain shift	Continuous non-contact observation of the froth phase
Online analyzers and spectroscopy	Elemental grade, Raman spectra, XRF composition	Seconds to minutes plus sample transport	Calibration, representativeness, blocked sampling lines, spectral interference	Near-real-time metallurgical reference or direct analytical measurement
Laboratory data	Chemical composition, particle size, mineralogy, liberation	Tens of minutes to hours	Sparse labels, manual entry, composite-sample timing, reporting delay	Reference labels, calibration, periodic validation
Equipment and actuator feedback	Valve position, pump speed, current, mode, interlock state	Sub-second to seconds	Deadband, backlash, saturation, commanded/actual mismatch	Context for separating process change from equipment limitations
Data-driven soft sensors	Estimated grade, recovery, state, anomaly score, uncertainty	Determined by input window and inference cycle	Concept drift, label leakage, extrapolation, unreported uncertainty	Continuous estimates of unmeasured or delayed variables

**Table 2:** Search and eligibility framework.

Component	Specification
Core process terms	“froth flotation” OR “flotation process” OR “flotation froth”
Sensing terms	sensor*, measurement, analyzer, spectroscopy, instrumentation, online monitoring, process data
Vision terms	machine vision, computer vision, froth image*, bubble size, froth velocity, segmentation, visual sensor
Learning terms	machine learning, deep learning, transformer, graph neural network, temporal model, self-supervised, soft sensor, data fusion
Industrial terms	SCADA, PLC, historian, LIMS, latency, domain shift, drift, uncertainty, industrial validation
Inclusion	Peer-reviewed English-language journal articles and full conference papers; 2021–2026; direct relevance to flotation sensing, state recognition, grade estimation, data quality, or industrial sensor integration
Exclusion	Ore sorting without flotation; purely chemical studies without measurement or modelling relevance; generic AI papers not used to support a review criterion; abstracts, patents, promotional reports, duplicate records, and unverifiable metadata

**Table 3:** Data-extraction and critical-appraisal framework.

Dimension	Extracted Evidence	Critical Question
Measurement definition	Measurand, units, reference method, spatial and temporal support	Is the model output a clearly defined process variable?
Acquisition reproducibility	Camera geometry, resolution, illumination, calibration, cleaning, synchronization	Can the measurement channel be reproduced and maintained?
Temporal integrity	Sampling rate, transport delay, aggregation window, chronological split	Do inputs and labels represent the same physical state?
Model validity	Baseline comparison, uncertainty, calibration, external validation	Is reported performance likely to generalize?
Operational robustness	Glare, contamination, missing data, drift, ore change, OOD behavior	Does the sensor fail safely under realistic disturbances?
Automation integration	Inference hardware, latency, historian/SCADA interface, quality flag, fallback	Can the estimate be used reliably by an industrial system?

**Table 4:** Dimensions and scoring rules of the proposed Sensor Readiness Index.

Dimension	Score 0	Score 1	Score 2
$\mathrm{Metrological}\;\mathrm{validity}\;(m_{i}$)	Setup not reproducible	Basic setup or calibration described	Geometry, illumination, resolution, quality control, and verification reported
$\mathrm{Temporal}\;\mathrm{integrity}\;(t_{i}$)	Temporal relation unclear	Partial delay handling or windowing	Event-time alignment, delay compensation, chronological split, and timestamp policy
$\mathrm{Validation}\;\mathrm{rigor}\;(v_{i}$)	Training/test separation unclear	Internal holdout or cross-validation	Chronological, inter-cell, inter-campaign, or external validation with baselines
$\mathrm{Operational}\;\mathrm{robustness}\;(r_{i}$)	No disturbance analysis	Limited augmentation or sensitivity test	Drift, contamination, lighting, OOD, uncertainty, missing data, and failure behavior evaluated
$\mathrm{Automation}\;\mathrm{integration}\;(g_{i})$	Offline model only	Real-time or advisory prototype	Verified latency, SCADA/historian interface, quality flag, model versioning, and fallback logic

**Table 5:** Thematic–chronological map of the evidence base.

Research Direction	2021	2022	2023	2024	2025	2026
PRISMA methodology and review context	[1,2,3]	[4]	[8]	[5]	[6,7,9]	—
Field instrumentation, online and laboratory data, data quality and datasets	—	[11]	[10,12,15]	[13,14]	—	—
Bubble segmentation, size, velocity, and image enhancement	—	[16]	[29,30,31]	[36,42,43,44]	[46,47]	[49,50]
Froth-state recognition and visual monitoring	[17,20]	[18,19,25,58]	[27,28,59]	[35,37,38,39,40,41]	[45]	—
Grade/ash-content and process-performance prediction; multimodal soft sensors	[21,22,23,24]	[26,51,53,56,57]	[32,52,55]	[33,34,54]	[48,60,61,63,64]	[62,65]
Industrial monitoring, digital twins, control, and optimization	[72,73]	[69]	[68,74,75]	[66,67,70,76,77,78]	[71,79]	—
General computer-vision, self-supervised learning, and video-analysis methods	[80,81,82,86,88]	[83,85,87]	[84]	[100]	—	—
Uncertainty, anomaly/OOD, and time-series analysis methods	[89,90,91,94,95,98]	—	[92,93,96,97,99]	—	—	—

Note. Sources are grouped by their principal role in this review; several publications relate to more than one direction. The table is intended for navigation of the evidence base and is not a mutually exclusive classification.

**Table 6:** Critical comparison of major flotation sensing tasks.

Task	Typical Outputs	Appropriate Primary Metrics	Required Secondary Evidence	Common Limitation
Bubble segmentation	Masks, instances, size distribution	IoU, Dice, boundary error, size-distribution error	Spatial calibration, unresolved-area fraction, annotation agreement	Good pixel score but biased physical size
Froth velocity	Pixel or physical velocity vectors	MAE/RMSE in velocity, directional error	Camera-motion compensation, frame-rate verification	Pixel displacement reported without physical scale
State recognition	Operating-condition class	Balanced accuracy, macro-F1, confusion matrix	Campaign-level split, class definition, OOD behavior	Random frame split and temporal leakage
Grade/ash soft sensing	Continuous metallurgical variable	MAE, RMSE, $R^{2}$, bias	Delay model, chronological validation, uncertainty calibration	Sparse or misaligned laboratory labels
Future-state prediction	Multi-step grade, velocity, state	Horizon-wise MAE/RMSE, coverage	End-to-end latency, recursive stability	Only one-step error reported
Multimodal fusion	Grade, state, anomaly score	Task metric plus ablation	Missing-channel test, modality quality, transfer test	Improvement reported without channel-failure analysis

**Table 7:** Representative comparison of predictive performance and published evidence of industrial sensor readiness.

Study	Task/Reported Result	SRI Profile M/T/V/O/A	Submitted Evidence of Readiness	Main Gaps and Industrial Interpretation
Zhou and He [35]	Industrial froth-state recognition; test accuracy 98.33%.	1/0/1/1/1 = 4/10	Real industrial images from gold–antimony flotation; K-fold splitting; image enhancement; ensemble model.	No split by independent campaigns is shown; calibrated uncertainty/OOD detection, end-to-end latency, a validity flag, and a verified PLC/SCADA fallback interface are absent. Very high accuracy alone does not imply high sensor readiness.
Bendaouia et al. [34]	ConvLSTM monitoring of Pb, Cu, Fe, and Zn; MAE = 4.52; a real-time architecture and GUI are presented.	1/1/1/1/1 = 5/10	Industrial froth video; explicit spatio-temporal model; real-time monitoring concept and deployment architecture.	External transferability, calibrated uncertainty/OOD detection, individual-channel failure, maintenance, and verified control handover are weakly disclosed. Deployment evidence is stronger than for a purely offline model but remains incomplete.
Hasidi et al. [66]	Industrial loop monitoring; 94% accuracy and 3 s response time over a one-month evaluation.	1/1/1/1/1 = 5/10	Industrial data; month-long evaluation; fast response; monitoring architecture and setpoint recommendations.	Temporal-leakage control, uncertainty calibration, fault modeling, channel absence, and closed-loop PLC validation are only partially disclosed. A lower overall accuracy than [35] is accompanied by stronger evidence of duration and integration
Ohenoja et al. [68]	Adaptive digital twin of a pilot flotation plant; no single headline accuracy reported; adaptation was tested under deliberate ore-type change and simulated sampling-rate/noise scenarios.	2/2/2/2/1 = 9/10	Defined XRF sampling and repeat analyses; 30 min sampling interval; hourly sliding windows; temporal alignment; ore-domain change; prediction/confidence intervals; sampling-rate and measurement-noise tests; near-real-time operation.	The phrase has been revised to “were not reported in the study” to avoid the ambiguous expression “not shown.; external industrial transferability is not confirmed. Strong readiness can be demonstrated through temporal, operational, and uncertainty-based validation even without a single high headline metric.

Note. The table does not treat the combination “low accuracy—high SRI” as a desirable opposite case. If performance on the target task falls below an acceptable level, the system cannot be considered a useful industrial sensor regardless of its degree of integration. The comparison separates predictive outcome from completeness of readiness evidence.

**Table 8:** Minimum reporting checklist for flotation machine-vision and multimodal sensing studies.

Category	Minimum Information
Process context	Ore/material, flotation stage, cell type, operating scale, campaign duration, principal disturbances
Measurement definition	Target variable, units, reference method, spatial/temporal support, sampling interval, delay model
Camera and acquisition	Camera/lens, field of view, working distance, resolution, frame rate, illumination, exposure, mounting, cleaning
Data construction	Number of campaigns, images/windows, class balance, label aggregation, missing-data treatment, quality flags
Validation	Grouping unit, chronological/external split, baselines, repeated runs, confidence intervals, leakage control
Robustness	Lighting, contamination, camera shift, missing channels, ore change, OOD detection, uncertainty calibration
Deployment	Hardware, full latency, interface, historian tags, model version, alarm/fallback policy, operator role

## Data Availability

No new data were created or analyzed in this study. Data sharing is not applicable to this article, as the review is based exclusively on the previously published literature cited in the References section.

## Supplementary Materials

The following supporting information can be downloaded at: https://www.mdpi.com/article/10.3390/s26175560/s1.
