# Supplementary material for: Multimodal Flotation Sensing: A Systematic Review of State Identification and Sensor Readiness
Source: Sensors (Basel). 2026 Sep 1;26(17):5560. doi: 10.3390/s26175560 (PMC13568359; doi:10.3390/s26175560)
Supplement: Supplementary file 1 [file sensors-26-05560-s001.zip › sensors-4491604-supplementary.pdf]

PRISMA 2020 Checklist

Section and Topic	Item #	Checklist item	Location where item is reported
**TITLE**			
Title	1	Identify the report as a systematic review.	Title page (article type: “Systematic Review”)
**ABSTRACT**			
Abstract	2	See the PRISMA 2020 for Abstracts checklist.	Abstract
**INTRODUCTION**			
Rationale	3	Describe the rationale for the review in the context of existing knowledge.	Section 1 (Introduction), paragraphs 1–4
Objectives	4	Provide an explicit statement of the objective(s) or question(s) the review addresses.	Section 1 (Introduction), research questions RQ1–RQ2; end of Introduction
**METHODS**			
Eligibility criteria	5	Specify the inclusion and exclusion criteria for the review and how studies were grouped for the syntheses.	Section 3.2 (Search strategy and eligibility criteria), Table 2
Information sources	6	Specify all databases, registers, websites, organisations, reference lists and other sources searched or consulted to identify studies. Specify the date when each source was last searched or consulted.	Section 3.1 (Review design and search period): Scopus and Web of Science Core Collection, searched through 30 June 2026; backward/forward citation tracking
Search strategy	7	Present the full search strategies for all databases, registers and websites, including any filters and limits used.	Section 3.2 (Search strategy and eligibility criteria), Table 2
Selection process	8	Specify the methods used to decide whether a study met the inclusion criteria of the review, including how many reviewers screened each record and each report retrieved, whether they worked independently, and if applicable, details of automation tools used in the process.	Section 3.2–3.3; Figure 4 (PRISMA-based study-selection workflow)
Data collection process	9	Specify the methods used to collect data from reports, including how many reviewers collected data from each report, whether they worked independently, any processes for obtaining or confirming data from study investigators, and if applicable, details of automation tools used in the process.	Section 3.3 (Data extraction and coding)
Data items	10a	List and define all outcomes for which data were sought. Specify whether all results that were compatible with each outcome domain in each study were sought (e.g. for all measures, time points, analyses), and if not, the methods used to decide which results to collect.	Section 3.3 (Data extraction and coding); Table 3 (data-extraction and critical-appraisal framework)
	10b	List and define all other variables for which data were sought (e.g. participant and intervention characteristics, funding sources). Describe any assumptions made about any missing or unclear information.	Section 3.3 (Data extraction and coding); Table 3
Study risk of bias assessment	11	Specify the methods used to assess risk of bias in the included studies, including details of the tool(s) used, how many reviewers assessed each study and whether they worked independently, and if applicable, details of automation tools used in the process.	Table 3 (data-extraction and critical-appraisal framework) and Table 4 (Sensor Readiness Index scoring rules), Section 3.3–3.4. A dedicated “Methodological appraisal” subsection explaining why formal clinical risk-of-bias tools do not apply is planned to be added to Section 3 upon revision.
Effect measures	12	Specify for each outcome the effect measure(s) (e.g. risk ratio, mean difference) used in	Not applicable – this is a descriptive/critical systematic

Section and Topic	Item #	Checklist item	Location where item is reported
		the synthesis or presentation of results.	review without pooled effect-size meta-analysis (see Section 5.8, Limitations of the review)
Synthesis methods	13a	Describe the processes used to decide which studies were eligible for each synthesis (e.g. tabulating the study intervention characteristics and comparing against the planned groups for each synthesis (item #5)).	Section 3.3–3.4 (narrative and critical synthesis by appraisal dimension, Table 3)
	13b	Describe any methods required to prepare the data for presentation or synthesis, such as handling of missing summary statistics, or data conversions.	Not applicable – no data conversions or missing-statistic handling required for narrative synthesis
	13c	Describe any methods used to tabulate or visually display results of individual studies and syntheses.	Section 4 (Results and Critical Synthesis); Tables throughout Section 4
	13d	Describe any methods used to synthesize results and provide a rationale for the choice(s). If meta-analysis was performed, describe the model(s), method(s) to identify the presence and extent of statistical heterogeneity, and software package(s) used.	Not applicable – no statistical meta-analysis performed (Section 5.8, Limitations of the review)
	13e	Describe any methods used to explore possible causes of heterogeneity among study results (e.g. subgroup analysis, meta-regression).	Not applicable – no statistical meta-analysis performed
	13f	Describe any sensitivity analyses conducted to assess robustness of the synthesized results.	Not applicable – no statistical meta-analysis performed
Reporting bias assessment	14	Describe any methods used to assess risk of bias due to missing results in a synthesis (arising from reporting biases).	Not applicable – no statistical meta-analysis performed; reporting bias not formally assessed given the narrative synthesis design (see Section 5.8)
Certainty assessment	15	Describe any methods used to assess certainty (or confidence) in the body of evidence for an outcome.	Not applicable – GRADE-type certainty assessment not performed; evidentiary strength is instead reported via the Sensor Readiness Index (Table 4, Section 3.4)
**RESULTS**			
Study selection	16a	Describe the results of the search and selection process, from the number of records identified in the search to the number of studies included in the review, ideally using a flow diagram.	Section 3.2–3.3; Figure 4 (PRISMA-based study-selection workflow)
	16b	Cite studies that might appear to meet the inclusion criteria, but which were excluded, and explain why they were excluded.	Not directly addressed – general exclusion criteria are specified in Table 2 (Section 3.2), but individual borderline studies that appeared eligible yet were excluded are not separately cited with study-specific reasons.
Study characteristics	17	Cite each included study and present its characteristics.	Reported narratively with in-text citations throughout Section 4 (Results and Critical Synthesis); Table 5 (Section 4) compares study evidence by sensing task rather than listing each of the 98 individual studies. No single consolidated table presenting characteristics of all included studies is currently provided.

PRISMA 2020 Checklist

Section and Topic	Item #	Checklist item	Location where item is reported
Risk of bias in studies	18	Present assessments of risk of bias for each included study.	Table 3 and Table 4 (Sensor Readiness Index), referenced throughout Section 4 where each topic is critically appraised against these dimensions.
Results of individual studies	19	For all outcomes, present, for each study: (a) summary statistics for each group (where appropriate) and (b) an effect estimate and its precision (e.g. confidence/credible interval), ideally using structured tables or plots.	Section 4 (Results and Critical Synthesis), narrative synthesis with in-text reporting of study-level evidence
Results of syntheses	20a	For each synthesis, briefly summarise the characteristics and risk of bias among contributing studies.	Section 4 (Results and Critical Synthesis)
	20b	Present results of all statistical syntheses conducted. If meta-analysis was done, present for each the summary estimate and its precision (e.g. confidence/credible interval) and measures of statistical heterogeneity. If comparing groups, describe the direction of the effect.	Not applicable – no statistical synthesis (meta-analysis) performed
	20c	Present results of all investigations of possible causes of heterogeneity among study results.	Not applicable – no statistical synthesis performed
	20d	Present results of all sensitivity analyses conducted to assess the robustness of the synthesized results.	Not applicable – no statistical synthesis performed
Reporting biases	21	Present assessments of risk of bias due to missing results (arising from reporting biases) for each synthesis assessed.	Not applicable – no statistical synthesis performed
Certainty of evidence	22	Present assessments of certainty (or confidence) in the body of evidence for each outcome assessed.	Not applicable – formal certainty-of-evidence framework (e.g., GRADE) not used; see Sensor Readiness Index, Table 4 (Section 3.4)
**DISCUSSION**			
Discussion	23a	Provide a general interpretation of the results in the context of other evidence.	Section 5 (Research Gaps and Deployment Framework)
	23b	Discuss any limitations of the evidence included in the review.	Section 5.8 (Limitations of the review)
	23c	Discuss any limitations of the review processes used.	Section 5.8 (Limitations of the review)
	23d	Discuss implications of the results for practice, policy, and future research.	Section 6 (Conclusions)
**OTHER INFORMATION**			
Registration and protocol	24a	Provide registration information for the review, including register name and registration number, or state that the review was not registered.	Section 3.1 (Review design and search period): this review was not registered in a public registry (e.g., PROSPERO)
	24b	Indicate where the review protocol can be accessed, or state that a protocol was not prepared.	Section 3.1: a formal review protocol was not prepared prior to conducting the review
	24c	Describe and explain any amendments to information provided at registration or in the protocol.	Not applicable – no registered protocol; no amendments to report
Support	25	Describe sources of financial or non-financial support for the review, and the role of the funders or sponsors in the review.	Funding statement (end of manuscript, before References)
Competing	26	Declare any competing interests of review authors.	Conflicts of Interest statement (end of manuscript,

PRISMA 2020 Checklist

Section and Topic	Item #	Checklist item	Location where item is reported
interests			before References)
Availability of data, code and other materials	27	Report which of the following are publicly available and where they can be found: template data collection forms; data extracted from included studies; data used for all analyses; analytic code; any other materials used in the review.	Data Availability Statement (end of manuscript, before References)

From: Page MJ, McKenzie JE, Bossuyt PM, Boutron I, Hoffmann TC, Mulrow CD, et al. The PRISMA 2020 statement: an updated guideline for reporting systematic reviews. BMJ 2021;372:n71. doi: 10.1136/bmj.n71. This work is licensed under CC BY 4.0. To view a copy of this license, visit https://creativecommons.org/licenses/by/4.0/
